# Roles of Nucleoporin RanBP2/Nup358 in Acute Necrotizing Encephalopathy Type 1 (ANE1) and Viral Infection

**DOI:** 10.3390/ijms23073548

**Published:** 2022-03-24

**Authors:** Jing Jiang, Yifan E. Wang, Alexander F. Palazzo, Qingtang Shen

**Affiliations:** 1 Department of Immunology, School of Basic Medical Sciences, Fujian Medical University, Fuzhou 350108, China; drenchedpross@foxmail.com; 2 Department of Biochemistry, University of Toronto, Toronto, ON M5G 1M1, Canada; yifa.wang@mail.utoronto.ca

**Keywords:** RanBP2, acute necrotizing encephalopathy type 1 (ANE1), viruses, cytokines

## Abstract

Ran Binding Protein 2 (RanBP2 or Nucleoporin358) is one of the main components of the cytoplasmic filaments of the nuclear pore complex. Mutations in the *RANBP2* gene are associated with acute necrotizing encephalopathy type 1 (ANE1), a rare condition where patients experience a sharp rise in cytokine production in response to viral infection and undergo hyperinflammation, seizures, coma, and a high rate of mortality. Despite this, it remains unclear howRanBP2 and its ANE1-associated mutations contribute to pathology. Mounting evidence has shown that RanBP2 interacts with distinct viruses to regulate viral infection. In addition, RanBP2 may regulate innate immune response pathways. This review summarizes recent advances in our understanding of how mutations in *RANBP2* contribute to ANE1 and discusses how RanBP2 interacts with distinct viruses and affects viral infection. Recent findings indicate that RanBP2 might be an important therapeutic target, not only in the suppression of ANE1-driven cytokine storms, but also to combat hyperinflammation in response to viral infections.

## 1. Introduction

Ran Binding Protein 2 (RanBP2), also known as Nucleoporin 358 KDa (Nup358), is one of the main components of the cytoplasmic filaments of the nuclear pore complex (NPC) [1]. RanBP2 was first identified in 1995 by both the Nishimoto and Blobel groups as a RanGTP binding protein [2,3] and contains several domains, including an N-terminal leucine-rich region [4], which is comprised of a tetratricopeptide repeat (TPR) segment followed by an alpha-solenoid segment, eight zinc finger motifs, four Ran binding domain (RBDs 1–4), and several phenylalanine-glycine (FG) repeats throughout the protein, as well as a C-terminal cyclophilin homology domain [3,5,6,7]. RanBP2 also contains a small ubiquitin-like modifier (SUMO) E3-ligase domain that covalently attaches the SUMO to various protein substrates. This E3 domain interacts with Ubiquitin Conjugating Enzyme 9 (Ubc9, the only known SUMO E2-conjugating enzyme in humans) and the SUMO-modified Ran GTPase activating protein (SUMO-RanGAP1) [8,9,10,11,12,13]. The formation of the tight RanBP2/SUMO-RanGAP1/Ubc9 complex at the cytoplasmic filaments of the NPC is essential for the SUMO E3-ligase activity of RanBP2 [14]. Aside from mediating sumoylation, RanBP2 has been implicated in many aspects of cellular processes, including nucleocytoplasmic transport [15,16,17,18], trafficking of photoreceptors [19,20], glucose metabolism [21], attachment of microtubules to kinetochores during mitosis [22,23,24,25], myogenesis [26,27], and mRNA metabolism [18,28,29], as well as microRNA-induced silencing [30,31,32,33]. Beyond the nuclear pore, RanBP2 also appears to form cytosolic aggregates, which may include annulate lamellae and/or some other biomolecular condensates [31,34,35]. In some cases, these structures appear to have mRNA and interact with other biomolecular condensates, such as stress granules and processing bodies (P-bodies) [31,35,36].

Mutations in RanBP2 are associated with acute-necrotizing encephalopathy type 1 (ANE1), a pediatric neurological disease that manifests as an overproduction of cytokines (known as a “cytokine storm”) after viral infection [37,38,39]. This can lead to seizures, coma, and high rate of mortality. However, how RanBP2 mutations promote the development of a cytokine storm in response to viral infection remains elusive.

## 2. RanBP2-Associated ANE1

Acute necrotizing encephalopathy (ANE) was first described by Mizuguchi in 1995. It is a rare disorder that is triggered by viral infections such as influenza or parainfluenza and causes a loss of consciousness, seizures, coma, and rapidly progressing encephalopathy [40]. Most cases of ANE occur sporadically and are non-familial and non-recurrent.

**Figure 1:**
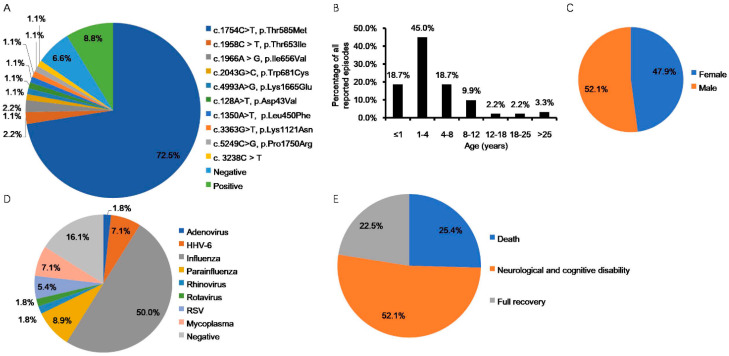
Statistical analysis of clinical information of ANE1 in 96 patients based on Table 1. (**A**) Percentage of *RANBP2* variants detected in all the reported cases. Negative indicates that no mutations were found in the coding region of the *RANBP2* gene in the familial or recurrent ANE patients, but the analyses did not preclude the possibility of an unidentified intronic mutation. Positive indicates that mutations were found in the coding region of the *RANBP2* gene, but the exact sites of the mutations were unclear. (**B**) Age distribution of all reported ANE1 episodes except for the ones without clear age information. (**C**) Gender distribution of all reported ANE1 cases. (**D**) Percentage of infectious agents detected in all reported episodes except for the ones without clear infectious agent information. (**E**) Percentage of outcomes in all the reported cases except for the ones without clear outcome information. List of abbreviations: ANE, acute necrotizing encephalopathy; RanBP2, Ran Binding Protein 2; HHV-6, human herpes virus-6; RSV, respiratory syncytial virus.

In 2009, Neilson et al. reported that familial, recurrent ANE cases are linked to missense mutations in the *RANBP2* gene involving c.1880C>T: p.Thr585Met, c.2085C>T: p.Thr653Ile, or c.2094A>G: p.Ile656Val by the old nomenclature, which are also known as c.1754C>T: p.Thr585Met, c.1958C>T:p.Thr653Ile, or c.1966A>G: p.Ile656Val by the Human Genome Variation Society (HGVS) preferred nomenclature [37,41]. Since then, ANE associated with RanBP2 mutations has been termed ANE1, which has also been renamed as infection-induced acute encephalopathy 3 (IIAE3) in the Online Mendelian Inheritance in the Man (OMIM) repository [38,42]. Aside from the mutations described above, several novel variations in the *RANBP2* gene, including c.2043G>C: p.Trp681Cys, c.4993A>G: p.Lys1665Glu, c.5249C>G: p.Pro1750Arg, c.3363G>T, p.Lys1121Asn, c.128A>T, p.Asp43Val, and c.1350A>T, p.Leu450Phe, have also been reported to be linked to ANE1 [38,39,43,44,45]. Though around 75% of familial, recurrent ANE cases are associated with heterozygous missense mutations in *RANBP2*, the penetrance of these dominant mutations was found to be only approximately 40% in the initial report, and half of ANE1 cases experience recurrent episodes [37,46]. It should be noted that these statistics are most reliable for the predominant disease associated mutation (Thr585Met), whereas the effects of other variants are less clear. ANE is known as a pediatric disease, as the majority of episodes occur in early childhood, though a few adult episodes have also been reported [37,47,48,49,50,51,52,53,54]. To date, 96 ANE1 patients have been reported in the medical literature (Table 1). Most ANE1 cases originated in North America and Europe; however, several ANE1 cases have also been reported in Asia, including Japan, South Korea, China, India, Malaysia, Saudi Arabia, and Iran (Table 1). As shown in Table 1 and Figure 1A, c.1754C>T: p.Thr585Met in the *RANBP2* gene is the most common ANE1-associated mutation (72.5% in all reported cases). Similar to ANE cases, ANE1 predominantly affects young children, especially ages 1–4 years old (45.0% in all reported ANE1 episodes) (Table 1 and Figure 1B) with the sex distribution between male (52.1%) and female (47.9%) being comparable (Table 1 and Figure 1C).

The majority of pathogens that trigger ANE1 are viruses, including the influenza virus (50.0%), parainfluenza virus (8.9%), human herpes virus 6 (7.1%), respiratory syncytial virus (5.4%), adenovirus (1.8%), rhinovirus (1.8%), and rotavirus (1.8%). In addition, it has been reported that bacteria mycoplasma (7.1%) can also induce ANE1, and only 16.1% of infectious workups in all reported ANE1 episodes were negative (Table 1 and Figure 1D).Of the reported ANE1 cases with clear outcome information, 25.4% died, 52.1% had sequela of neurological and cognitive disability, and 22.5% experienced full recovery (Table 1 and Figure 1E).

## 3. Diagnosis of ANE1

The diagnostic criteria of ANE and ANE1 have been well described previously [37,41,47,83]. Briefly, ANE patients present an acute encephalopathy within 1–3 days following the onset of a febrile illness, which frequently show deterioration of consciousness, seizures, and culminate in coma. Radiologically, ANE results in symmetrical and multifocal brain lesions specifically involving bilateral thalami, and oftentimes periventricular white matter, internal capsule, putamen, brainstem, and cerebellum. Other clinical and laboratory findings of ANE include an increase in cerebrospinal fluid (CSF) proteins, but absence of CSF pleocytosis, and oftentimes an increase in serum transaminases with normal ammonia. In addition, the diagnosis of ANE should exclude the possibilities of infectious, metabolic, autoimmune, and toxic diseases [37,47]. However, criteria suggestive of ANE1 require any one of the following criteria, aside from the findings of ANE: (I) prior episodes of encephalopathy following fever, (II) familial history of ANE or acute neurological symptoms, (III) additional characteristic lesions in the central nervous system (CNS) as detected by MRI imaging. These lesions tend to occur in the medial temporal lobes, insular cortices, claustra, external capsule, amygdalae, hippocampi, mammillary bodies, and spinal cord [37,41,47,62].

## 4. Pathogenesis of ANE1: Roles of RanBP2 in ANE1

Although it has been known that missense mutations in *RANBP2* are genetic factors that contribute to ANE1, their actual role in the pathogenetic mechanisms of this disease remains unclear.

As described previously, RanBP2 has been implicated in several different cellular processes, including energy maintenance in neurons by perhaps regulating microtubule and/or mitochondrial function [21,37,84,85]. Therefore, one speculative explanation is that mutations in RanBP2 cause mitochondrial metabolic disorders in response to viral infection. Indeed, mice that are heterozygous null for the *RANBP2* gene show metabolic defects, especially in glucose metabolism [21]. In addition, certain SNPs in the gene encoding the mitochondrial enzyme carnitine palmitoyl transferase 2 (CPT2) have been linked to ANE, suggesting a further link between mitochondrial metabolism and this disease [86]. ANE mutations may alter the association of RanBP2 with the mitochondrial cytochrome c oxidase assembly protein COX11 [87]. Despite these observations, the precise mechanism of how RanBP2 mutations could possibly alter mitochondrial metabolism is not understood.

Another plausible theory, which is the most widely accepted, is that mutations in RanBP2 directly trigger a “cytokine storm” after infection. Previous reports showed that ANE patients had elevated concentrations of cytokines including proinflammatory cytokines such as interleukin 6 (IL6), tumor necrosis factor-α (TNF-α), IL10, interferon γ (IFNγ), soluble tumor necrosis factor (sTNF) receptor, and IL15 in their CSF and plasma [88,89,90,91,92,93,94,95]. The resulting elevated levels of proinflammatory cytokines induce the injury of vascular endothelial cells and increase the blood–brain barrier (BBB) permeability. These cytokines may then infiltrate into the central nervous system to induce apoptosis of glial cells and neurons, as well as stimulate glial cells to release more cytokines, and ultimately cause neurological sequelae [96,97,98,99,100,101,102,103]. Alternatively, cytokines may be produced within the CNS by microglial cells. Indeed, mice that have a conditional knockout of the *RANBP2* gene in retinal ganglion cells show increased microglial activation [104], suggesting a role of RanBP2 in the regulation of innate immune cells.

Recently, we documented that RanBP2 promotes the microRNA-mediated suppression of ANE1-associated cytokines, such as IL6 and TNF-α [30]. We found that RanBP2 sumoylates Argonaute proteins thereby promoting their ability to silence the *IL6* mRNA. Our data is consistent with the model that Argonaute proteins first interact with the *IL6* mRNA in the nucleus and accompany the mRNA as it traverses the nuclear pore. Then just after the completion of nuclear export, these Argonautes are sumoylated by RanBP2. This sumoylation event stabilizes Argonaute onto the *IL6* mRNA, thereby enforcing its silencing. Our results suggest that, under certain circumstances, ANE1-associated mutations alter microRNA-dependent silencing, which eventually causes a massive secretion of cytokines. In agreement with this idea, RanBP2-depletion has been shown to reduce the number of P-bodies, which are cytoplasmic foci that have been implicated in microRNA-silencing [31,105]. Moreover, it has been recently shown that ANE1 mutations disrupt the association between RanBP2 and the Argonaute binding partner GW182 [32]. Despite this, a form of RanBP2 containing three of the ANE1-associated mutations (Thr585Met, Thr653Ile, and Ile656Val) rescued Argonaute1-dependent silencing of the *IL6* mRNA in human osteosarcoma cells [30]. Thus, if ANE1 is due to the mis-regulation of Argonaute activity and thus cytokine overproduction, these mutations may only have an effect in either pathologically relevant cell types and/or viral infected cells.

There may be other ways in which mutations in RanBP2 trigger cytokine storms, possibly through the over activation of innate immune pathways. It is known that the production of cytokines requires the activation of innate immune pathways. These sense viral infections and stimulate downstream transcription factors, including interferon (IFN)-regulatory factor 3 (IRF3), IRF7, nuclear factor kappa-light-chain-enhancer of activated B cells (NF-κB, consisting of p65 and p50), and signal transducer and activator of transcription (STAT) proteins [106]. In general, the hyperactivation of these signals can cause cytokine storms. In some cases, RanBP2 was found to inhibit NF-κB-mediated innate immune responses [107]. Other studies showed that the sumoylation of the signal transducers, such as NF-κB or STAT1, inhibits their activation [108,109,110]. However, it remains unclear whether these sumoylation events are triggered by the SUMO E3-ligase RanBP2 and whether this is altered by ANE1-associated mutations. This will require further studies that focus on the interplay between RanBP2 and/or its ANE1-associated mutations, with innate immune signaling pathways.

Aside from the genetic influence of *RANBP2* mutations, additional environmental factors, such as pathogen infection, are required for ANE1 development [47,83]. As discussed above, most ANE1 cases were induced by various viral infections (see Table 1 and Figure 1). It is possible that ANE1-associated mutations in *RANBP2* alter the viral infection process by impacting the interactions between RanBP2 and either viral proteins or anti-viral host factors. Indeed, RanBP2 has been reported to be required for the replication of a variety of viruses (summarized in Table 2), which will be discussed in the next section.

## 5. The Interplay between RanBP2 and Viruses

### 5.1. Herpes Simplex Viruses

Herpes simplex viruses (HSVs) are double-stranded DNA viruses that belong to *Herpesviridae*. HSVs include herpes simplex virus type 1 and 2 (HSV-1 and HSV-2). Most humans are infected by HSV-1, and part of them present clinical symptoms, such as cold sores. In 2008, Hofemeister and O’Hare showed that HSV infection reduces the amount ofO-glycosylation in RanBP2, which may affect NPC function [111]. It has also been reported that the HSV-1 capsid-tethered tegument protein VP1/2 (encoded by the viral gene *UL36*) interacts with RanBP2, thus allowing capsids to attach to the surface of the nuclear envelope [112]. In addition, the HSV-1 minor capsid protein pUL25 interacts with CAN/Nup214 and hCG1, two other components of the cytoplasmic filaments of the nuclear pore besides RanBP2. This is thought to initiate viral DNA uncoating and promote the release of viral DNA into the nucleoplasm. Since RanBP2 is in close proximity to Nup214, it is believed that they constitute a platform for the attachment of HSV-1 capsids to the cytoplasmic face of the nuclear pore [106,113]. Although RanBP2 is known to play a critical role in docking HSV capsids to the NPC, there are few reports of HSV infection triggering ANE1.

### 5.2. Adenoviruses

Adenoviruses are a family of nonenveloped double-stranded DNA viruses that infect a broad range of vertebrate species and cause respiratory, gastrointestinal, ocular, and urogenital diseases. Strunze et al. showed that during adenovirus infection, RanBP2 interacts with the kinesin-1 heavy-chain (KIF5C) to promote the transport of NPC components (RanBP2, Nup214, and Nup62) away from the nucleus and into the cytoplasm. This transport is thought to disrupt the nuclear envelope and promote the transport of viral DNA from viral capsids, which are docked onto NPCs, to the exposed nucleoplasm [114].

In another study, the authors proposed a model whereby RanBP2 serves as an assembly platform where the adenovirus genome becomes coated with nuclear transport receptors, facilitating its nuclear import [115]. To date, only one report has linked adenovirus infections to ANE1 [57].

### 5.3. Vaccinia Virus

Vaccinia virus (VACV) is a double-stranded DNA virus that belongs to the family of *Poxviridae*. VACV is known as a live, naturally attenuated vaccine used for eradicating smallpox caused by variola virus, another member of the *Poxviridae* family [130]. VACV entirely replicates in the cytoplasm, specifically in membrane-delimited vesicles, termed viral factories [131]. Viral factories resemble mini-nuclei, in that they contain nuclear-pore-like structures consisting of several nucleoporins, including Nup62 and RanBP2 [116,132]. Although RanBP2 depletion does not significantly affect VACV DNA replication, it significantly reduces the size and number of viral factories and the viral yield of VACV [116]. Indeed, RanBP2 is essential for recruiting Nup62 and translation factors, such as eukaryotic translation initiation factor eIF4E, to viral factories. Thus, RanBP2 plays a critical role in VACV life cycles. Whether *Poxviridae* viruses can cause ANE1 remains unknown as smallpox has been eradicated.

### 5.4. Papillomaviruses

Papillomaviruses are small, nonenveloped, icosahedral DNA viruses with an 8 kb circular double-stranded DNA genome, which belong to the family *Papillomaviridae* [133]. Papillomaviruses encode two proteins, E1 and E2, that are required for viral DNA replication in host cell nuclei [134,135]. The post-translational modifications of E1, including phosphorylation and sumoylation, play critical roles in modulating its function [136,137,138,139,140,141,142,143]. Although RanBP2 was found to bind to bovine papillomavirus type 1 E1, it was not required for E1 sumoylation [117]. Instead, this interaction is believed to contribute to the import of the E1 protein to the nucleus [117]. Curiously, RanBP2 does not interact with the E1 from human papillomavirus 11, suggesting that this mechanism for E1 nuclear import is not universal.

### 5.5. Severe Acute Respiratory Syndrome-Coronavirus 2

Severe acute respiratory syndrome-coronavirus 2 (SARS-CoV2) is an enveloped, single-stranded, positive-sense RNA virus with a genome of approximately 30-kb, which belongs to beta-coronavirus of the family *Coronaviridae* [144,145]. SARS-CoV2 is highly pathogenic in humans, has infected over 417 million individuals globally, and caused 5.8 million deaths by mid-February 2022 according to the coronavirus disease 2019 (COVID-19) map from Johns Hopkins University. Most COVID-19-associated deaths are due to the development of cytokine storms that manifest mostly in the lungs of patients.

SARS-CoV2 proteins interact with many human proteins including distinct nuclear transport receptors and nucleoporins [146]. SARS-CoV2 ORF6 was observed to interact with RanBP2 and other nuclear pore filament proteins [118]. ORF6 appears to modulate nucleo-cytoplasmic trafficking and may affect the export of cytokine mRNAs [118,146,147,148]. Interestingly, one recent study showed that SARS-CoV2 infection downregulates the expression level of RanBP2 [119].

The link between RanBP2 and cytokine storms suggest that it may play a role in the development of the COVID-19 disease [106,149,150,151,152,153,154]. There are additional links between COVID-19 and ANE. First, deceased COVID-19 patients have a high rate of neuropathology that resembles ANE [155]. Second, several case studies indicated that COVID-19 can also lead to ANE-like cytokine storms in the brain [156,157,158,159,160]; however, whether these patients bear mutations in RanBP2 or whether ANE1 mutations affect the response to SARS-CoV2 infection remains to be determined.

### 5.6. Human Rhinovirus

Human rhinovirus (HRV) is a nonenveloped, single-stranded, positive-sense RNA virus belonging to the family *Picornaviridae*, which infects the upper respiratory tract and also leads to the majority of asthma exacerbations [161,162,163]. During HRV infection, nucleoporins including RanBP2, Nup153, and Nup214 were shown to be degraded by the 3C protease (3C^pro^) and its precursor, 3CD, both encoded by HRV. The degradation of these nucleoporins is thought to disrupt NPCs, enhance nuclear envelope permeability, and alter nucleocytoplasmic trafficking [106,120]. To date, there is only one report of rhinovirus infection triggering ANE1 [77]. Despite this, whether ANE1 mutations in RanBP2 affect how the body responds to HRV infection is unknown.

### 5.7. Hepatitis C Virus and Japanese Encephalitis Virus

Both hepatitis C virus (HCV) and Japanese encephalitis virus (JEV) belong to members of the *Flaviviridae* family, which are single-stranded, positive-sense RNA viruses.

Like VACV, HCV replicate in viral factories [121,164]. These contain nuclear pore-like structures, which consist of RanBP2, Nup153, Nup155, Nup98, and Nup53. The pore-like structures play a critical role in the transport of macromolecules into the interior of viral factories to facilitate viral replication and assembly, as well as viral immune evasion [121,122]. Interestingly, it has been observed that HCV infection increases both mRNA and protein levels of RanBP2 [121]. This suggests that RanBP2 contributes to the replication and assembly of HCV and may also help viral immune evasion.

Interestingly, JEV infection also increased RanBP2 expression; however, the depletion of RanBP2 significantly increases JEV replication [123]. To date, it remains unclear whether these two viruses trigger ANE1.

### 5.8. Influenza Virus

The influenza virus is a single-stranded, negative-sense RNA virus, which belongs to the *Orthomyxoviridae* family. There are four type species of influenza virus, including Influenza A, B, C, and D. Influenza A is highly contagious to humans and causes seasonal flu epidemics [165]. To date, Influenza A virus (IAV) is known to be one of the most common pathogens leading to ANE1. However, it remains unclear how Influenza A interacts with RanBP2 and its ANE1 mutations. Future investigations focusing on the molecular mechanism of their interaction will help determine the exact pathogenesis of Influenza A-induced ANE1.

### 5.9. Human Immunodeficiency Virus Type-1 (HIV-1)

Human immunodeficiency virus type-1 (HIV-1) is an enveloped, single-stranded, positive-sense RNA virus that belongs to the genus *Lentivirus* of the *Retroviridae* family. HIV-1 attacks the body’s immune system and can cause acquired immunodeficiency syndrome (AIDS) at the late stage of its infection. Upon entry into host cells, the HIV-1 virion releases viral RNA genomes into the cytoplasm, and then the single-stranded RNA genome is reverse transcribed into complementary DNA by reverse transcriptase encoded by HIV-1. The resulting viral DNA, which is associated with viral proteins, such as integrase, and host co-factors in the form of preintegration complex (PIC), is then imported into the nucleus and integrated into the host genome [166,167], allowing the virus to evade the host immune response and become latent for an indeterminate amount of time [168]. It has been observed that the C-terminal cyclophilin domain (Cyp) of RanBP2 interacts with the Cyclophilin A (CypA) binding loop of HIV-1 capsid (CA), and this interaction facilitates the nuclear import of HIV-1 PIC and viral infection [124,125,126,127,128]. Interestingly, Rasaiyaah et al. showed that while HIV-1 CA mutant N74D is impaired in associating with the cleavage and polyadenylation specificity factor subunit 6 (CPSF6), the CA mutant P90A fails to interact with RanBP2 and cyclophilin A, and both mutants trigger type-1 interferon (IFN-I) production and induce an antiviral state by activating innate immune signaling pathways. This may indicate that the interaction between host factors, such as RanBP2, and the HIV-1 CA protein helps to evade innate immune sensors by restricting viral complementary DNA production to the nuclear pore and facilitating its rapid import into the nucleus [129]. Although RanBP2 plays a critical role in the HIV-1 life cycle, it remains unclear whether HIV-1 infection could trigger ANE1.

## 6. Conclusions

Taken together, this review presents the progress that has been made towards understanding how dominant mutations in *RANBP2* cause ANE1 and summarizes recent advances in understanding the interactions between RanBP2 and distinct viruses. It has been known that viruses are the major environmental triggers for ANE1 in individuals with certain *RANBP2* missense mutations. However, it remains unclear how ANE1 mutations in *RANBP2* contribute to disease development. These mutations may directly impact the viral infection process, or act downstream in the modulation of immune cells, immune activation and/or cytokine production.

As detailed above, RanBP2 interacts with different viruses and plays a role in viral infection. Despite this, we have yet to have any reports that show whether/how ANE1-associated mutations alter RanBP2-virus interactions, or whether all the viruses interacting with RanBP2, as discussed above, cause ANE1. Future studies focusing on the interactions between ANE1-associated mutations, viruses, and host antiviral innate immune responses are needed to clarify the molecular mechanisms that cause this disease. Ultimately, this work will also help develop novel therapeutic strategies against ANE1 and similar pathological states caused by viral-induced cytokine storms.

## Figures and Tables

**Table 1:** Clinical information of ANE1 in 96 patients.

Study, (*n* Cases)	Country of Origin	Age	Gender (F:M)	Infectious Agents	Recurrent Cases (*n*, %)	RanBP2 Mutation (*n*, %)	Outcome (*n*, %)	References
Neilson et al., 2009 (32) ^a^	USA, Australia, UK, Switzerland, Denmark, Greece, Germany	Range ^b^ (*n*, %) <1 y: (1/32, 3%) 1~3 y: (3/32, 9%) 8~12 y: (1/32, 3%) 13~18 y: (1/32, 3%) >25 y: (1/32, 3%) N/A: (25/32, 78%)	15:17	Influenza (8 epi), Parainfluenza (1 epi), Mycoplasma (1 epi)	14/32, 44%	c.1880C>T, p.Thr585Met (30/32, 94%); c.2085C>T, p.Thr653Ile (1/32, 3%); c.2094A>G, p.Ile656Val (1/32, 3%)	D (2/32, 6%), NCD (5/32, 16%), FR (4/32, 13%), N/A (21/32, 65%)	[37,46,55,56]
Loh et al., 2010 (1)	UK	18 m (1st epi) 3 y 11 m (2nd epi)	0:1	Adenovirus (1st epi), Influenza (2nd epi)	1/1, 100%	3238C>T (1/1, 100%)	FR (1/1, 100%)	[57]
Marco et al., 2010 (3)	USA (Eastern Europeandescent)	C1:11m C2:23m C3:18 m (1st epi), 8 y (2nd~4th epi)	0:3	C1: HHV-6 C2: Influenza C3: N/A	1/3, 33%	C1-3: negative (3/3, 100%) ^c^	D (3/3, 100%)	[58]
Gilson et al., 2011 (1)	UK	5 y	1:0	N/A	0	c.1754C>T; p.Thr585Met (1/1, 100%)	NCD (1/1, 100%)	[59]
Howayyer et al., 2011 (1)	Canada (Canadian Aboriginal Cree descent)	34 m (1st epi), 5 y (2nd epi)	0:1	Negative (1st epi), Influenza (2nd epi)	1/1, 100%	g.33868A>G p.Ile656Val (1/1, 100%)	NCD (1/1, 100%)	[60]
Lönnqvist et al., 2011 (6)	Finland	C1:12 y C2:9 m (1st epi), 6 y (2nd epi) C3-6:7 m to 6 y	3:3	N/A	2/6, 33% ^d^	c.1880C>T, p.Thr585Met (6/6, 100%)	FR (1/6, 17%) NCD (5/6, 83%)	[61]
Bergaminoet al., 2012 (1)	Italy	5m(1st epi), 18 m (2nd epi), 26 m (3rd epi)	0:1	Rotavirus (1st epi), RSV (2nd epi)	1/1, 100%	c.1880C>T, p.Thr585Met (1/1, 100%)	FR (1/1, 100%)	[62]
Lee et al., 2012 (1)	Korea	12 m (1st epi), 22 m (2nd epi)	0:1	Influenza (1st and 2nd epi)	1/1, 100%	Negative ^c^ (1/1, 100%)	NCD (1/1, 100%)	[63]
Wolf et al., 2013 (1)	Switzerland	36 y	1:0	Negative	0	c.1880C>T, p.Thr585Met (1/1, 100%)	FR (1/1, 100%)	[64]
Denier et al., 2014 (3)	France	C1: 1 y (1st epi), 2 y (2nd epi), 9 y (3rd epi) C2:6 m (1st epi), 2 y (2nd epi), 6 y (3rd epi), 23 y (4th epi) C3: 5 y	2:1	C1: N/A C2: Influenza (2nd and 4th epi) C3: N/A	2/3 (67%)	C1: N/A (1/3, 33%) C2–3:c.1754C>T, p.Thr585Met (2/3, 67%)	C1: D (1/3, 33%) C2–3: NCD (2/3, 67%)	[65]
McSwiney et al., 2014 (1)	Australia	3 y	1:0	Influenza	0	Positive (1/1, 100%)	NCD (1/1, 100%)	[66]
Di Meglio et al., 2014 (2)	France (Tunisian descent)	C1: 9 m (1st epi), 9 y (2nd epi) C2: 9 m	1:1	N/A	1/2, 50%	C1-2:c.1880C>T, p.Thr585Met (2/2, 100%)	C1: D (1/2, 50%), C2: NCD (1/2, 50%)	[67]
Anand et al., 2015 (1)	UK	28 m	1:0	Influenza	0	c.2085C>T, p.Thr653Ile (1/1, 100%)	FR (1/1, 100%)	[68]
Bloch et al., 2015 (2)	Switzerland	C1:10 y C2:40 y	1:1	C1-2:Influenza	0	C1-2:c. 1754 C>Tp.Thr585Met (2/2, 100%)	C1-2: NCD (2/2, 100%)	[69]
Singhet al., 2015 (2)	UK	C1:2 y 7 m C2:1 y 4 m	2:0	C1: N/A C2: negative	0	C1-2:c.1880C>T: p.Thr585Met (2/2, 100%)	C1: NCD (1/2, 50%) C2: FR (1/2, 50%)	[55]
Sell et al., 2016 (2)	Germany	C1:10 m C2:19 m (1st epi), 22 m (2nd epi), 36 m (3rd epi)	0:2	C1: HHV-6 C2: N/A	1/2, 50%	C1: c. 1754 C>T, p.Thr585Met (1/2, 50%) C2: c.2043G>C, p.Trp681Cys (1/2, 50%)	C1: NCD (1/2, 50%) C2: D (1/2, 50%)	[38]
Sondhi et al., 2016 (1)	India	3.5 y (1st epi), 3 y 11 m (2nd epi),	1:0	Negative (1st and 2nd epi)	1/1, 100%	c.1754 C>T, p.Thr585Met (1/1, 100%)	NCD (1/1, 100%)	[41]
Nishimura et al., 2016 (2)	Japan	C1:3 y 5 m C2:4 y 8 m	0:2	C1: N/A C2: Influenza	0	Negative (2/2, 100%) ^c^	C1: D (1/2, 50%) C2: N/A (1/2, 50%)	[70]
Lee et al., 2017 (2)	South Korea	C1:2 y C2:12 m	2:0	C1: Negative C2: Mycoplasma	0	C1: N/A (1/2, 50%), C2:c.1754C>T, p.Thr585Met (1/2, 50%)	C1: D (1/2, 50%) C2: NCD (1/2, 50%)	[71]
Alawadhi et al., 2018 (1)	Canada	6 y	1:0	Negative	0	c.4993A>G, p.Lys1665Glu (1/1, 100%)	NCD (1/1, 100%)	[45]
Howard et al., 2018 (2)	Mexico	C1:5 y C2:17 m	1:1	C1-2: Influenza	0	C1-2:c.1754C>T, p.Thr585Met (2/2, 100%)	C1: FR (1/2, 50%) C2: D (1/2, 50%)	[72]
Isikay et al., 2018 (1)	Turkey	12 y (1st epi), 14 y (2nd epi)	0:1	N/A	1/1, 100%	c.1754C>T, p.Thr585Met (1/1, 100%)	FR (1/1, 100%)	[73]
Soriano-Ramos et al., 2018 (1)	Spain	7 m (1st epi), 19 m (2nd epi), 24 m (3rd epi), 10 y (4th epi)	0:1	N/A (1st~3rd epi), Influenza (4th epi)	1/1, 100%	c.1754C>T, p.Thr585Met (1/1, 100%)	NCD (1/1, 100%)	[74]
Kelly et al., 2019 (1)	Australia	15 m (1st epi), 27 m (2nd epi), 5 y (3rd epi), 22 y (4th epi)	1:0	Influenza (3rd epi)	1/1, 100%	c.1754C>T, p.Thr585Met (1/1, 100%)	NCD (1/1, 100%)	[75]
Bashiri et al., 2020 (2) ^e^	Saudi Arabia	N/A	N/A	N/A	N/A	c.3363G>T, p.Lys1121Asn (1/2, 50%), c.128A>T, p.Asp43Val (1/2, 50%)	N/A (2/2, 100%)	[43]
Chew et al., 2020 (3)	Malaysia	C1:11y C2:4y6m C3:2y6m	3:0	C1: Negative C2: Parainfluenza C3: Parainfluenza, Mycoplasma	0	C1-3: c.1754C>T, p.Thr585Met (3/3, 100%)	C1-2: NCD (2/3, 67%) C3: FR (1/3, 33%)	[76]
Chowet al., 2020 (2)	China	C4:15 m C5:9 m (1st epi), 22 m (2nd epi)	1:1	C4: N/A C5: Rhinovirus(1st epi), Parainfluenza(2nd epi)	1/2, 50%	C4-5: c.1754C>T, p.Thr585Met (2/2, 100%)	C4-5: D (2/2, 100%)	[77]
Huang et al., 2020 (1)	China	11 m	0:1	HHV-6	0	c.1754C>T, p.Thr585Met (1/1, 100%)	N/A (1/1, 100%)	[78]
Iyer et al., 2020 (3)	India	C1:11m(1st epi), 15 m (2nd epi), 20 m (3rd epi) C2:9m C3:13m(1st epi), 24 m (2nd epi)	1:2	C1-3: N/A	2/3, 67%	C1:c.5249C>G, p.Pro1750Arg ^f^ (1/3, 33%), C2–3: N/A (2/3, 67%)	C1–3: D (3/3, 100%)	[39]
Xavier et al., 2020 (1)	Portugal	5y	0:1	Influenza	0	c.1754C>T, p.Thr585Met (1/1, 100%)	NCD (1/1, 100%)	[79]
Hartley et al., 2021 (2)	USA	C1: 9 m (1st epi), 2 y 9 m (2nd epi) C2: 6 y	2:0	C1: HHV-6 (1st epi) C2: negative	1/2, 50%	C1: c.1754C>T; p.Thr585Met (1/2, 50%) C2: c.1350A>T, p.Leu450Phe (1/2, 50%)	C1–2: NCD (2/2, 100%)	[44]
Ohashi et al., 2021 (1)	Japan	1 y 7 m (1st epi), 1 y 9 m (2nd epi)	0:1	Parainfluenza (1st epi), RSV (2nd epi)	1/1, 100%	c.1754C>T, p.Thr585Met ^g^ (1/1, 100%)	NCD (1/1, 100%)	[80]
Paktinat et al., 2021 (3)	Iran	C1: 7 y (1st epi), 9 y (2nd epi) C2: 4 y C3: 4 y (1st epi), 6 y (2nd epi)	1:2	C1-3: N/A	2/3, 67%	C1–2: c.1754C>T, p.Thr585Met (2/3, 67%) C3: N/A (1/3, 33%)	C1: NCD (1/3, 33%) C2: FR (1/3, 33%) C3: D (1/3, 33%)	[81]
Chatur et al., 2022 (7)	Canada	Range, 4 m–10 y	3:4	Influenza (3), Mycoplasma (1), RSV (1) N/A (2)	3/7, 43%	Positive (7/7, 100%)	D (1/7, 14%), NCD (4/7, 57%), FR (2/7, 29)	[82]

List of abbreviations: ANE, acute necrotizing encephalopathy; RanBP2, Ran Binding Protein 2; F, female; M, male; y, year; m, month; CSF, cerebrospinal fluid; C1, Case1; NR, normal; D, death; FR, full recovery; NCD, neurological and cognitive disability; epi, episode(s); RSV, respiratory syncytial virus; HHV-6, human herpes virus-6; N/A, not available. ^a^ Certain cases in the study have been reported in other studies [46,56]. ^b^ The ages shown here only indicate the age of the first episode of each case. ^c^ No mutations in the coding region of the *RANBP2* gene were found in the familial or recurrent ANE patients, but this analysis did not preclude the possibility of an unidentified intronic mutation. ^d^ The report showed both Cases 2 and 5 have 2 episodes; however, whether the other patients were recurrent cases were unclear. ^e^ This retrospective study showed novel missense heterozygous variants of RanBP2 (c.3363G>T, p.Lys1121Asn and c.128A>T, p.Asp43Val) in two ANE1 patients. ^f^ The patients also had mutation in the *Carnitine Palmitoyl Transferase 2 (CPT2)* gene (c.365C>T, p.S122F). ^g^ The patient also had mutation in *CPT2* (c.1055T>G, p.Phe352Cys).

**Table 2:** The interaction between RanBP2 with distinct viruses.

Virus Group (Baltimore Classification)	Virus Family	Virus	Consequence(s) (References)
I (dsDNA viruses)	*Herpesviridae*	HSV-1	Reducing the levels of O-glycosylated RanBP2 [111] Facilitating HSV-1 capsid attachment to the nuclear surface [112,113]
	*Adenoviridae*	Adenoviruses	Disrupting the nuclear envelope and facilitating the transport of viral DNA into the nucleus [114,115]
	*Poxviridae*	VACV	Maintaining the size and number of viral factories and facilitating viral yield of VACV [116]
	*Papillomaviridae*	BPV	Contributing to the import of viral protein E1 to the nucleus in bovines [117]
IV ((+) ssRNA viruses)	*Coronaviridae*	SARS-CoV-2	Downregulating the expression level of RanBP2and might facilitate the development of “cytokine storms” in most severe patients of COVID-19 [106,107,118,119]
	*Picornaviridae*	HRV	Degrading RanBP2 and disrupting nuclear envelope permeability and nucleocytoplasmic trafficking [120]
	*Flaviviridae*	HCV	Increasing mRNA and protein levels of RanBP2, and might contribute to HCV replication, assembly, and viral immune evasion [121,122]
		JEV	Increasing RanBP2 expression and the knockdown of RanBP2 can increase JEV replication [123]
V ((−) ssRNA viruses)	*Orthomyxoviridae*	IAV	Unknown
VI (ssRNA-RT viruses)	*Retroviridae*	HIV-1	Facilitating the rapid import of HIV-1 pre-integration complex into nucleus to evade innate immune sensors and facilitating viral infection [124,125,126,127,128,129]

List of abbreviations: RanBP2, Ran Binding Protein 2; HSV-1, herpes simplex virus type 1; VACV, Vaccinia virus; BPV, bovine papillomavirus; SARS-CoV-2, severe acute respiratory syndrome-coronavirus 2; COVID-19, coronavirus disease 2019; HRV, human rhinovirus; HCV, hepatitis C Virus; JEV, Japanese encephalitis virus; IAV, influenza A virus; HIV-1, Human immunodeficiency virus type-1.

## Data Availability

Not applicable.
